# A bibliometric analysis of the current state and future directions of osteoporosis pharmacological treatment

**DOI:** 10.3389/fmed.2025.1622425

**Published:** 2025-09-24

**Authors:** Xianxian Zhou, Hua Xiong, Dexi Hu

**Affiliations:** Department of General Practice, Yiyang Central Hospital, Yiyang, China

**Keywords:** osteoporosis, medication, bibliometrics, visual analysis, citespace, VOSviewer

## Abstract

**Introduction:**

Osteoporosis is a major health threat, particularly with the aging population in China. Medication remains a cornerstone of management, and bibliometric analysis can provide insights into current research status and future directions.

**Methods:**

Relevant literature from the Science Citation Index Core Collection (2015–2024) was analyzed using bibliometric methods. Visual maps were generated with Citespace 6.3R3 and VOSviewer 1.6.19 to assess research trends and hotspots.

**Results:**

A total of 2,738 publications were included, showing a steady growth in research since 2015. The United States led in output, with the University of Toronto as the most productive institution. Brandi, Maria Luisa, and Kanis JA were the most influential authors, while Osteoporosis International and The Journal of Bone and Mineral Research were the most cited journals. Key themes included extracellular vesicles, romosozumab, bisphosphonates, and breast cancer, with recent attention on targeted drug delivery, treatment efficacy, and medication management. Emerging keywords from 2022 to 2023, such as exosomes, inflammation, and osteogenic differentiation, reflected advances in therapeutic mechanisms and clinical applications.

**Conclusion:**

Future research will likely emphasize targeted drug delivery, clinical efficacy and safety, and molecular targeted therapies, with the development of new anti-osteoporosis drugs remaining a key focus.

## 1 Introduction

With global population aging, osteoporosis has become a major public health concern. Characterized by reduced bone density and microarchitectural deterioration, it markedly increases fracture risk, especially among the elderly (1). Hip and spinal compression fractures not only raise mortality but also impair quality of life, often leading to disability and long-term care needs (2, 3). Epidemiological studies indicate that over 20% of individuals above 60 and more than 50% of those over 80 are affected (4), highlighting its substantial clinical and societal burden.

Pharmacological therapy remains central to osteoporosis management. Agents such as bisphosphonates, selective estrogen receptor modulators, and parathyroid hormones effectively slow bone loss and reduce fracture risk (5). Despite the clinical importance of these therapies, bibliometric studies in this field are scarce. Existing work largely emphasizes individual drugs or treatment strategies, with limited comprehensive, quantitative evaluation of the overall research landscape.

This study therefore conducts a systematic bibliometric analysis of publications on osteoporosis drug treatment. By assessing publication volume, collaborations, journal distribution, and research hotspots, we aim to map current knowledge, identify emerging trends, and outline future research directions. Such an approach not only highlights scientific progress but also provides valuable guidance for researchers and clinicians in the field.

## 2 Materials and methods

### 2.1 Data source and search methodology

On February 3, 2025, two researchers independently performed a literature search. In the event of any disagreement, a third researcher will make the final decision. The search was conducted using the following search formula: “(TS = (Drug Therapy or Chemotherapy or Chemotherapies or Pharmacotherapy or Pharmacotherapies or Therapy), Drug or Drug Therapies or Therapies, Drug) AND TS = (Osteoporosis or Osteoporoses or Osteoporosis, Age-Related or Osteoporosis, Age Related or Age-Related Osteoporosis or Age-Related Osteoporoses or Age Related Osteoporosis or Osteoporoses, Age-Related or Bone Loss, Age-Related or Age-Related Bone Loss or Age-Related Bone Losses or Bone Loss, Age Related or Bone Losses, Age-Related or Osteoporosis, Senile or Osteoporoses, Senile or Senile Osteoporoses or Senile Osteoporosis or Osteoporosis, Involutional or Osteoporosis, Post-Traumatic or Osteoporosis, Post-Traumatic or Post-Traumatic Osteoporoses or post-traumatic Osteoporosis)”. The search covered literature published between January 2015 and December 2024, limited to English-language articles and reviews. Materials such as “Editorial Material,” “Letter,” and “Meeting Abstract” were excluded. The Web of Science Core Collection database was used for the subject word search, yielding a total of 2,738 relevant articles. All retrieved documents were exported as “Full Records and Cited Literature” in plain text format and downloaded. CiteSpace (version 6.3R3) was then employed to remove duplicates, and the cleaned dataset was saved.

### 2.2 Data analysis

In this research, the overall volume of publications by authors and countries/regions was assessed using WPS software. Data visualization was performed with GraphPad Prism 9.5 (USA), while the world map was concurrently created with ArcMap 10.8. Subsequently, the complete dataset of literature was imported into CiteSpace 6.3R3 and VOSviewer 1.6.19 for further analysis. In CiteSpace 6.3R3, the time slice was set to 1 year, and the threshold was defined as “the first 50 nodes per slice.” Co-citations of authors and institutions were examined using VOSviewer 1.6.19, generating the corresponding visual maps.

### 2.3 Main observation indicators

A visual examination was performed on the co-citation patterns and keywords associated with countries/regions, institutions, authors, and journal articles, with the objective of uncovering the current state of research, key areas of focus, and future trends in this domain.

## 3 Results

### 3.1 Publication analysis by year

**FIGURE 1:**
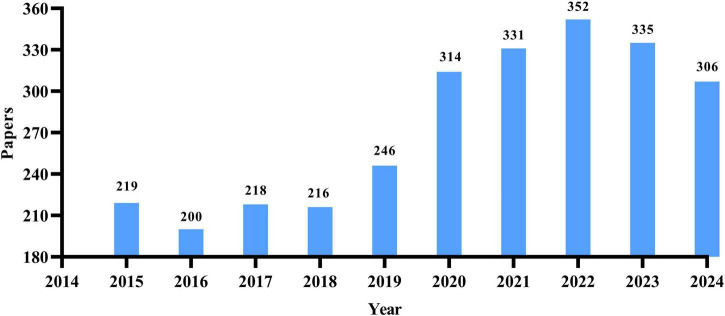
Annual distribution map of the number of research papers on drug treatment for osteoporosis in the Web of Science Core Collection database.

Among the 2,738 works meeting the inclusion criteria, there were 1,920 research papers and 818 review articles. As shown in Figure 1, from 2015 to 2024, there has been a notable overall increase in the annual publication rate of research papers related to osteoporosis drug treatment. The period from 2015 to 2018 represented a low plateau in publications. However, starting in 2019, there has been a transition into a phase of rapid growth, peaking at 352 articles in 2022. While there has been a slight decline in the number of publications since 2023, the figure has remained above 300, indicating sustained research interest in this area, albeit shifting from rapid growth to a focus on structural optimization.

### 3.2 Analysis of publishing countries

**FIGURE 2:**
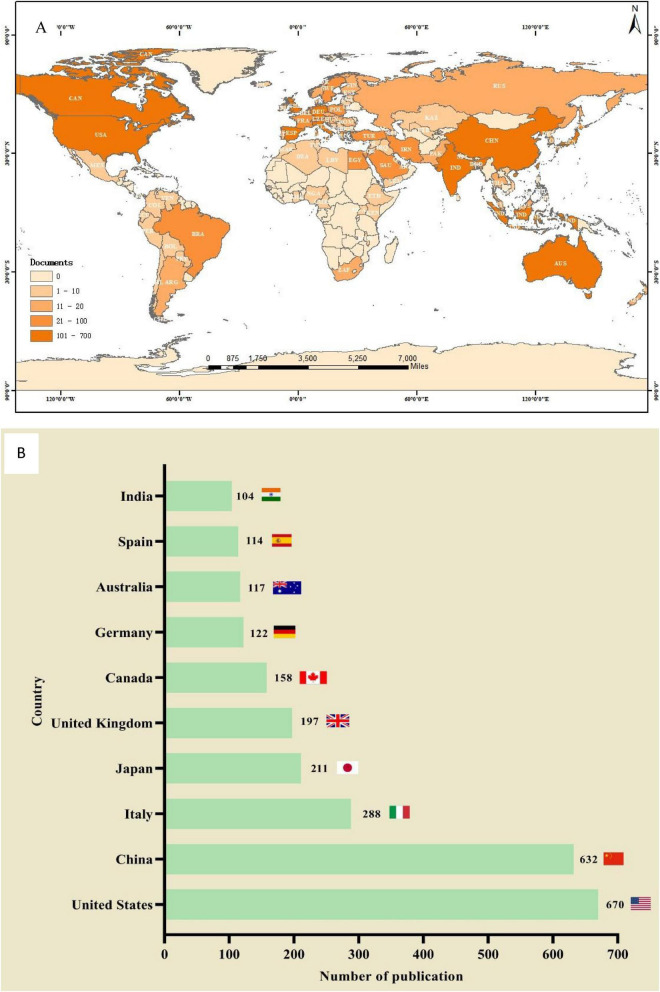
The total number of literatures published by countries/regions on the research of drug treatment for osteoporosis in the Web of Science Core Collection database. **(A)** Geographic distribution. **(B)** The number of publication count in countries with more than 100 articles published.

**FIGURE 3:**
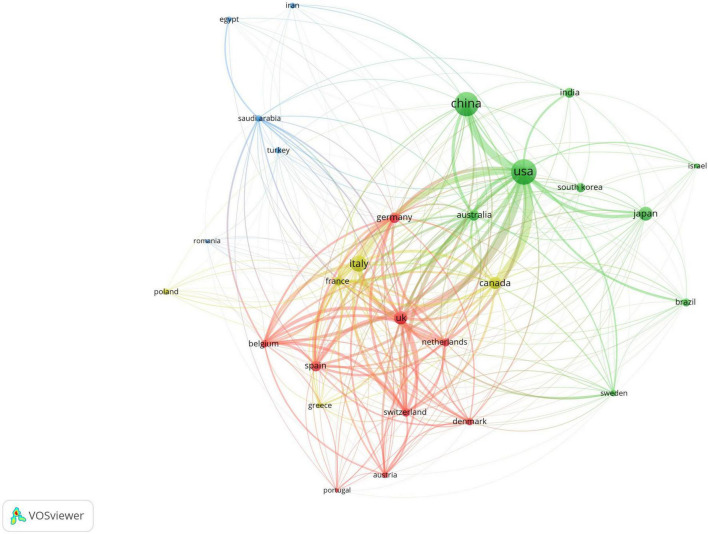
Co-occurrence chart of the number of papers published by various countries in the field of drug treatment for osteoporosis research in the Web of Science Core Collection database.

A total of 93 countries worldwide have contributed to scholarly publications in the area of pharmacological osteoporosis research (Figure 2A). The United States leads in publication volume with 670 articles, representing 37.8% of the overall total. Following the U.S. are China (632 articles), Italy (288 articles), Japan (211 articles), and the United Kingdom (197 articles), as shown in Figure 2B. The collaborative relationships and co-occurrence network among these countries are depicted in Figure 3. Notably, there are significant academic exchanges and collaborations between the United States and China, as well as between the United States and the United Kingdom.

### 3.3 Institutional publication analysis

**FIGURE 4:**
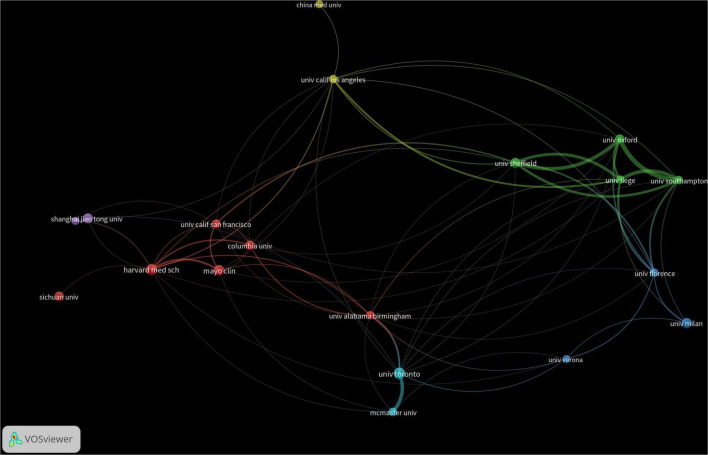
Co-occurrence chart of the number of institutional publications in the field of drug treatment for osteoporosis research in the Web of Science Core Collection Database.

A total of 4,254 institutions worldwide have engaged in research on drug treatments for osteoporosis, with only two institutions publishing more than 40 papers. Among these, the University of Toronto has the highest publication count at 48 papers, followed by Harvard Medical School in the U.S. with 43 papers, and the Mayo Clinic with 38 papers. In terms of citation counts, the Australian Catholic University leads with 3,042 citations, trailed by Harvard Medical School with 2,827 citations and the Mayo Clinic with 2,756. For detailed data, refer to Table 1. Regarding academic collaboration, the University of Oxford (UK) and the University of Southampton, as well as the University of Oxford and the University of Sheffield, exhibit particularly strong cooperation. Conversely, collaboration among other high-output institutions requires further enhancement. Detailed information can be found in Figure 4.

**TABLE 1:** The top 10 institutions in the Web of Science database in terms of the number of publications and the number of citations on drug treatment of osteoporosis.

Rank	Institution	Country	Number of publications	Number of citations	Institution	Country	Number of citations
1	University of Toronto	Canada	48	1,017	Australian Catholic University	Australia	3,042
2	Harvard Medical School	United States	43	2,827	Harvard Medical School	United States	2,827
3	Mayo Clinic	United States	38	2,756	Mayo Clinic	United States	2,756
4	Shanghai Jiao Tong University	China	36	1,011	The University of Sheffield	The United Kingdom	2,500
5	University of Milan	Italy	34	750	University of Liège	Belgium	2,410
6	University of Sheffield	The United Kingdom	34	2,500	Oregon Health and Science University	United States	2,368
7	Sichuan University	China	32	723	University of Oxford	The United Kingdom	2,186
8	Columbia University	United States	31	2,158	University of California, Los Angeles	United States	2,166
9	University of Oxford	United States	31	2,186	University of Southampton	The United Kingdom	2,159
10	University of California, San Francisco	United States	29	1,144	Columbia University	United States	2,158

### 3.4 Examination of published authors

**FIGURE 5:**
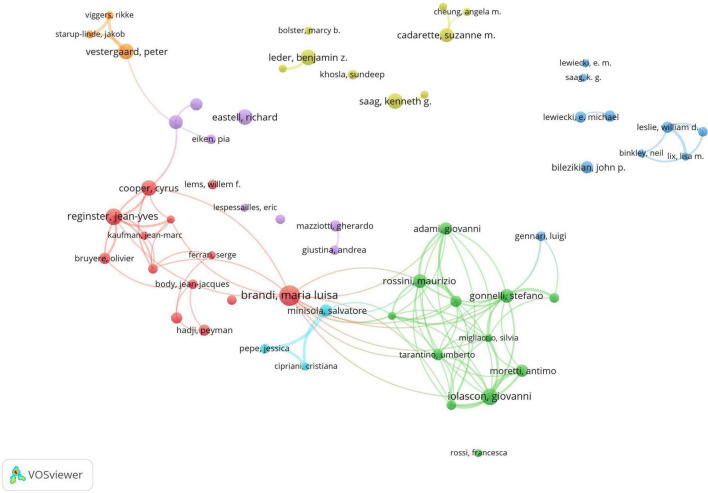
Co-occurrence chart of co-authors in the field of drug treatment for osteoporosis research in the Web of Science Core Collection Database.

A total of 15,800 authors have contributed to research in drug treatments for osteoporosis. The leading authors in terms of published articles are Brandi, Maria Luisa (14 papers), Iolascon, Giovanni (11 papers), and Reginster, Jean-Yves (11 papers). Co-cited authors, referring to scholars cited in multiple publications, total 73,337. In terms of citations, the top three co-cited authors are Kanis, JA (948 citations), Black, DM (771 citations), and Cosman, F (625 citations). Detailed information is available in Table 2. As for academic collaboration, notable partnerships include those between Iolascon, Giovanni and Moretti, Antimo; Rossini, Maurizio and Gatti, Davide. The collaboration network can be viewed in Figure 5.

**TABLE 2:** The top 10 co-authors and co-cited authors of drug therapy for osteoporosis.

Rank	Co-author	Number of publications	Number of citations	Co-cited authors	Number of citations
1	Brandi, Maria Luisa	14	525	Kanis, J. A.	948
2	Iolascon, Giovanni	11	181	Bblack, D. M.	771
3	Reginster, Jean-Yves	11	580	Cosman, F.	625
4	Cooper, Cyrus	10	534	Cummings, S. R.	560
5	Eastell, Richard	10	335	Mcclung, M. R.	414
6	Leder, Benjamin, Z.	10	714	Eastell, R.	361
7	Saag, Kenneth, G.	10	495	Miller, P. D.	353
8	Su, Jiacan	10	363	Bone, H. G.	344
9	Vestergaard, Peter	10	93	Khosla, S.	340
10	Cadarette, Suzanne, M.	9	74	Reid, I. R.	322

### 3.5 Examination of published journals

**FIGURE 6:**
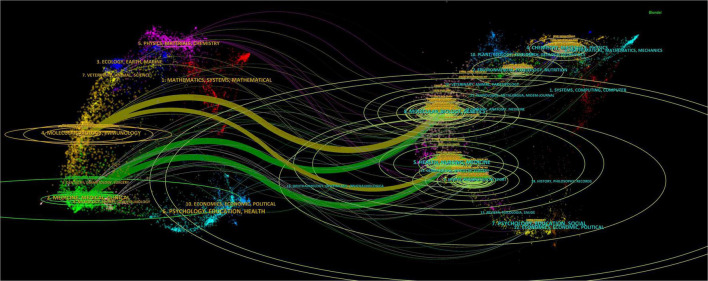
The double-image superposition of journals in the field of drug treatment for osteoporosis research in the Web of Science Core Collection database. The cited journals are on the left and the cited journals are on the right. The colored curves represent the citation paths.

A total of 2,738 papers were published across 1,065 academic journals. Among these, “Osteoporosis International” received the highest number of citations, totaling 4,854. Other journals with significant citation counts include the “Journal of Bone and Mineral Research,” “Lancet,” “Bone,” and “Lancet Diabetes & Endocrinology.” Four of the top ten journals have impact factors exceeding 5 (see Table 3). Of the 12,912 co-cited journals, five have been cited more than 3,000 times. The specific data can be found in Table 4. “Journal of Bone and Mineral Research” has the highest co-citation count (8,011), followed by “Osteoporosis International” and “Bone.” The dual maps of the journals illustrate the distribution of academic subjects (see Figure 6). Two primary green citation paths were identified, showing that studies in medical, pharmaceutical, and clinical journals were mainly cited by research in health, nursing, medical, and molecular, biological, and genetic journals. Additionally, two yellow citation paths highlight that studies published in molecular, biological, and immunological journals were predominantly cited by research in health, nursing, medicine, and molecular, biological, and genetic journals. These citation paths illustrate the flow of knowledge between disciplines, indicating how clinical and pharmaceutical findings influence broader biomedical research and healthcare-related fields. Such connections are significant because they reflect the interdisciplinary nature of osteoporosis pharmacological research, demonstrating both the clinical relevance of molecular studies and the translational impact of clinical research on basic biomedical science.

**TABLE 3:** The top 10 journals of drug therapy for osteoporosis in Web of Science.

Rank	Journal	Total number of citations	IF (2023)	JCR division (2023)	Country
1	Osteoporosis International	4,854	4.2	Q2	The United Kingdom
2	Journal of Bone and Mineral Research	2,710	5.1	Q1	United States
3	Lancet	2,538	98.4	Q1	The United Kingdom
4	Bone	1,246	3.5	Q2	United States
5	Lancet Diabetes and Endocrinology	1,050	44	Q1	The United Kingdom
6	International Journal of Molecular Sciences	967	4.9	Q2	Switzerland
7	Frontiers in Pharmacology	858	4.4	Q2	Switzerland
8	Cochrane Database of Systematic Reviews	784	8.8	Q1	The United Kingdom
9	Archives of Osteoporosis	734	3.1	Q2	The United Kingdom
10	BMJ Open	652	2.4	Q2	The United Kingdom

**TABLE 4:** The top 10 co-cited journals of drug therapy for osteoporosis in Web of Science.

Rank	Co-cited journals	Co-citations	IF (2023)	JCR division (2023)	Country
1	Journal of Bone and Mineral Research	8,011	5.1	Q1	United States
2	Osteoporosis International	7,611	4.2	Q2	The United Kingdom
3	Bone	4,796	3.5	Q2	United States
4	Journal of Clinical Endocrinology and Metabolism	3,850	5	Q1	United States
5	New England Journal of Medicine	3,373	96.2	Q1	United States
6	Lancet	1,933	98.4	Q1	The United Kingdom
7	Jama-Journal of the American Medical Association	1,725	63.1	Q1	United States
8	PLoS One	1,581	2.9	Q1	United States
9	Calcified Tissue International	1,488	3.3	Q2	United States
10	Journal of Clinical Oncology	1,361	42.1	Q1	United States

### 3.6 Co-citation analysis of literature

The co-occurrence analysis of cited references was performed using CiteSpace, and the ten most frequently cited references were identified (Table 5). Among these, four were clinical studies involving the use of two biological agents in the treatment of osteoporosis (references 2, 3, 4, and 8 in the table), three focused on clinical practices and management strategies for drug treatments of postmenopausal osteoporosis (references 5, 6, and 7 in the table), and two examined clinical trials on bisphosphonates for osteoporosis treatment (references 9 and 10 in the table). Additionally, one article offered a comprehensive review of the epidemiology, etiology, and risk management of osteoporosis (reference 1 in the table).

**TABLE 5:** The top 10 most cited articles on drug therapy research for osteoporosis in the Web of Science database.

Rank	Title of the cited literature	Publication time	Number of citations	Centrality	Main contribution
1	Osteoporosis	2019	89	0.02	A comprehensive overview of the epidemiology, etiology, and risk management of osteoporosis.
2	Romosozumab or Alendronate for Fracture Prevention in Women with Osteoporosis	2017	76	0.05	For the first time, a comparison was made between the novel osteoporosis drug romosozumab and the traditional drug alendronate, finding that romosozumab is more effective in preventing fractures in postmenopausal women, significantly reducing the risk of new vertebral fractures, clinical fractures, and hip fractures.
3	Romosozumab Treatment in Postmenopausal Women with Osteoporosis	2016	71	0.05	One year of romosozumab treatment significantly increased spinal and hip bone mineral density in postmenopausal women, and continued to reduce the risk of vertebral fractures over the next 1.5 years, providing a new and effective option for the treatment of osteoporosis.
4	10 years of denosumab treatment in postmenopausal women with osteoporosis: results from the phase 3 randomized FREEDOM trial and open-label extension	2017	68	0.05	The main contribution of this study lies in evaluating the safety and efficacy of denosumab in postmenopausal women with osteoporosis over a period of 10 years, demonstrating that long-term use can still significantly reduce the risk of fractures and maintain bone density.
5	European guidance for the diagnosis and management of osteoporosis in postmenopausal women	2020	67	0.05	A comprehensive diagnostic and management framework was provided for the evaluation and treatment of osteoporosis in postmenopausal women, particularly focusing on risk classification and treatment strategies for patients with low, moderate, and high fracture risk.
6	Clinician’s Guide to Prevention and Treatment of Osteoporosis	2014	67	0.02	A comprehensive guide for clinicians on the prevention, risk assessment, diagnosis, and treatment of osteoporosis was provided, with particular emphasis on its applicability to women and men over the age of 50, as well as how to effectively manage osteoporosis through both pharmacological and non-pharmacological approaches.
7	Pharmacological Management of Osteoporosis in Postmenopausal Women: An Endocrine Society Clinical Practice Guideline	2019	66	0.1	Evidence-based clinical practice recommendations for the pharmacological management of osteoporosis in postmenopausal women were provided, including risk assessment, treatment options, and consideration of patient preferences.
8	Vertebral Fractures After Discontinuation of Denosumab: A *Post Hoc* Analysis of the Randomized Placebo-Controlled FREEDOM Trial and Its Extension	2018	66	0.04	The study aimed to evaluate the risk of new or worsening vertebral fractures after discontinuation of denosumab, particularly the changes in the risk of multiple vertebral fractures, through the FREEDOM trial and its extended retrospective analysis.
9	Managing Osteoporosis in Patients on Long-Term Bisphosphonate Treatment: Report of a Task Force of the American Society for Bone and Mineral Research	2016	63	0.05	Management recommendations were provided for patients on long-term bisphosphonate therapy for osteoporosis, including the duration of medication use and strategies for reassessment after discontinuation, to balance efficacy and potential side effects.
10	Atypical Subtrochanteric and Diaphyseal Femoral Fractures: Second Report of a Task Force of the American Society for Bone and Mineral Research	2014	57	0.06	A systematic review of the epidemiology, pathophysiology, and medical management of atypical femoral fractures was conducted, presenting diagnostic and classification criteria, and emphasizing the association between long-term bisphosphonate (BPs) use and these fractures.

The most cited article (89 citations) was written by Compston et al. (6) and published in The Lancet in 2019. This manuscript addressed the optimization of osteoporosis management strategies in clinical settings, focusing on the enhancement of risk assessment tools (such as FRAX) and the standardized interpretation of bone mineral density tests. It also emphasized evidence-based choices of anti-resorption drugs (bisphosphonates) and bone-promoting medications (e.g., teriparatide), advocating for individualized treatment approaches. The study proposed differentiated intervention thresholds and sequential drug treatment strategies based on patients’ risk levels. This research is significant as it combines advances in molecular biology with clinical practice needs, offering a vital evidence base for the revision of the WHO’s osteoporosis prevention and treatment guidelines, and supporting the development of a precision medicine model based on fracture risk stratification.

The most central reference, with a centrality score of 0.12, was an article by Bone, HG (7) published in the Journal of Clinical Endocrinology and Metabolism in 2018. This study assessed the effectiveness and safety of an 18-month treatment with abalopeptide (ABL) or placebo (PBO), followed by 24 months of alendronate sodium (ALN) in postmenopausal women with osteoporosis. The results demonstrated that the ABL/ALN combination reduced the risk of new vertebral fractures by 84% (0.9 vs. 5.6%) compared to the PBO/ALN group, with reductions in non-vertebral and major osteoporotic fractures ranging from 39 to 50%. Additionally, bone mineral density increased across several sites (lumbar vertebrae, total hip, and femoral neck) by 14.9, 5.5, and 6.3%, respectively, compared to baseline. The study confirmed that the combined ABL and ALN therapy could sustain long-term anti-fracture efficacy, providing an effective treatment strategy for high-risk patients, with significant clinical implications.

### 3.7 Research hotspot analysis

#### 3.7.1 Co-citation timeline of references

**FIGURE 7:**
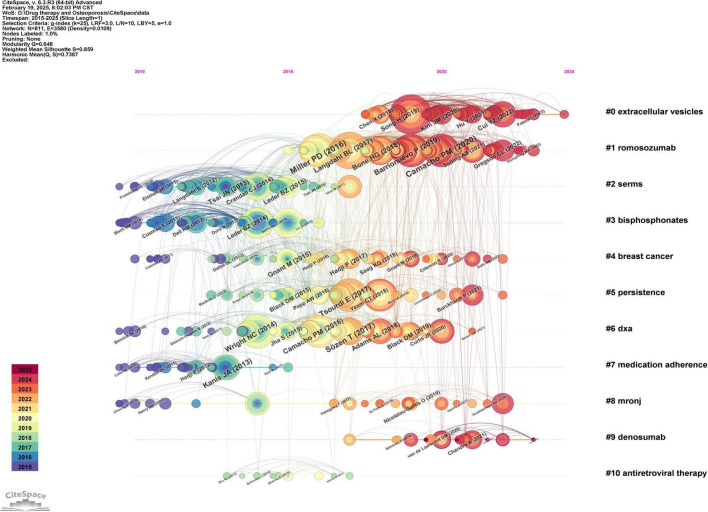
Co-cited time chart of the references in the field of drug treatment for osteoporosis research in the Web of Science Core Collection database.

A co-citation timeline of references was created using Citespace software, as illustrated in Figure 7. The references within the same cluster are arranged along the timeline based on their publication dates. Topics such as “extracellular vesicles,” “romosozumab,” “selective estrogen receptor modulators,” “bisphosphonates,” and “breast cancer” are most frequently cited. The literature associated with the clusters “extracellular vesicles,” “romosozumab,” and “desumab” appears to be at the forefront of the field, as indicated by the timing of their release.

#### 3.7.2 Co-occurrence analysis of keywords

**FIGURE 8:**
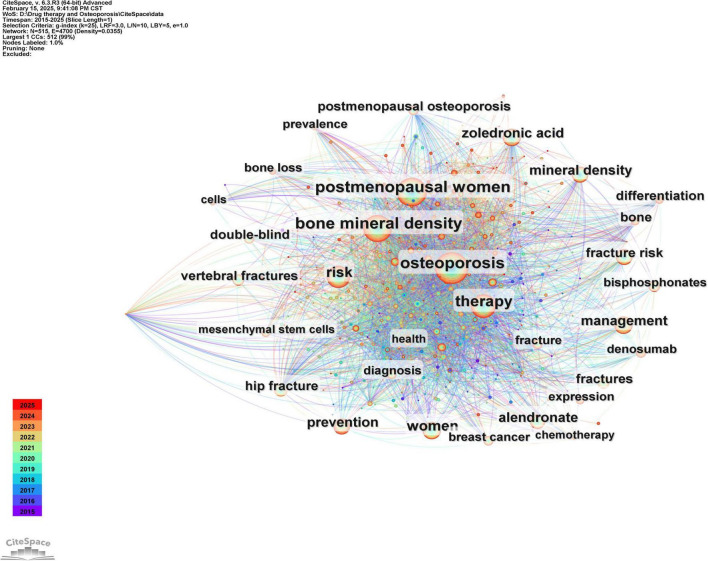
Co-occurrence graph of key words in the field of drug treatment for osteoporosis research in the Web of Science Core Collection Database.

**FIGURE 9:**
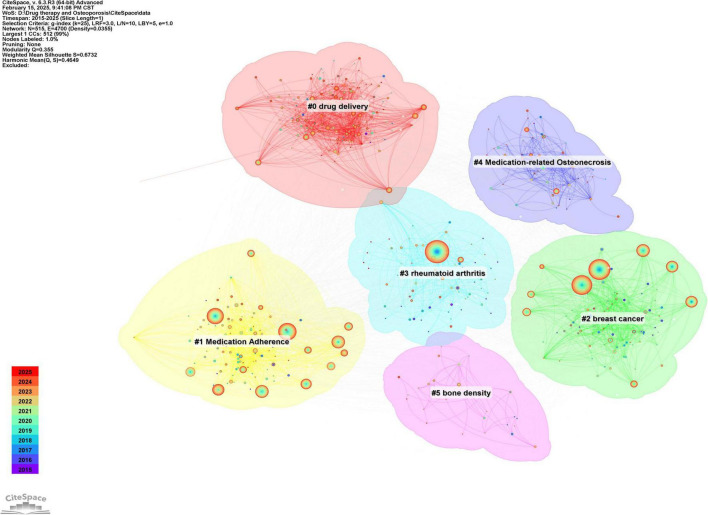
Keyword clustering map of the research field of drug treatment for osteoporosis in the Web of Science Core Collection database.

Keywords serve as concise representations of the core themes of an article, typically reflecting the primary focus of the research area. Keywords that are both highly frequent and centrally located often highlight the research topics with the most current influence. A co-occurrence analysis of keywords across all the literature revealed that the primary research themes are centered around postmenopausal osteoporosis, bone mineral density, fracture risk, and bisphosphonates, with strong correlations among these terms, as shown in Figure 8 and Table 6. To further identify emerging research fields, a keyword clustering analysis was performed, and a clustering map was generated, resulting in six distinct clusters as depicted in Figure 9. The size of a cluster corresponds to the number of keywords it contains, with the clusters ordered as follows based on label size: #0 Drug delivery, #1 Medication compliance, #2 Breast cancer, #3 Rheumatoid arthritis, #4 Drug-related osteonecrosis, and #5 Bone mineral density. These clusters are interrelated, continually evolving, and not isolated from each other. The color of each cluster region indicates the time of the first co-citation, with red denoting earlier clusters and green and blue representing those emerging later.

**TABLE 6:** Top 20 keywords of drug therapy for osteoporosis research in Web of Science.

Rank	Key words	Frequency	Centrality
1	Osteoporosis	546	0.02
2	Bone mineral density	527	0.02
3	Postmenopausal women	515	0.03
4	Therapy	398	0.02
5	Risk	314	0.01
6	Women	260	0.01
7	Zoledronic acid	243	0.02
8	Management	233	0.01
9	Mineral density	231	0.02
10	Prevention	227	0.01
11	Alendronate	191	0.01
12	Fracture risk	162	0.02
13	Hip fracture	146	0.02
14	Fractures	138	0.01
15	Vertebral fractures	136	0.01
16	Bone	133	0.02
17	Postmenopausal osteoporosis	130	0.01
18	Double-blind	129	0.02
19	Differentiation	128	0.03
20	Bisphosphonates	121	0.01

### 3.8 Emergent words and future trends

**FIGURE 10:**
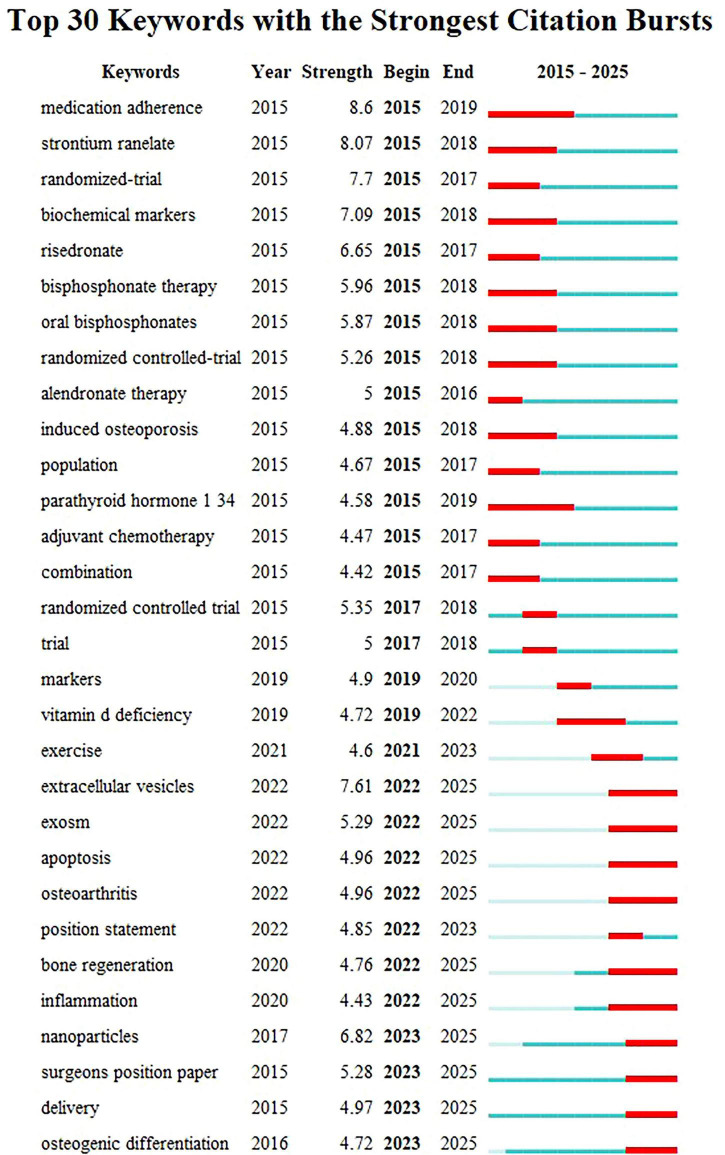
The keyword emergence chart in the field of drug treatment for osteoporosis research in the Web of Science Core Collection database.

Emergent words are keywords that show a significant increase in frequency over a short period, reflecting shifts in the focus of a research field. Based on the keyword clustering analysis mentioned earlier, the emergent word functionality in Citespace was used to explore the latest trends in osteoporosis drug treatment, resulting in the emergent word map shown in Figure 10. Thirty emergent keywords were identified. The red area on the right side of the figure marks the time period during which these keywords became prominent. The analysis reveals that from 2015 to 2019, keywords such as “medication compliance” and “bisphosphonates” emerged consistently, indicating their strong influence in the research during this period. Since 2019, terms like “markers,” “vitamin D deficiency,” and “clinical trials” have become more frequent, signaling a growing focus on basic research and clinical exploration with deeper research methods (2019–2021). Furthermore, keywords such as “extracellular vesicles,” “exosomes,” “apoptosis,” “osteoarthritis,” “bone regeneration,” “inflammation,” “nanoparticles,” “surgical guidelines,” “delivery,” and “osteogenic differentiation” have remained prominent. This suggests that the molecular targeted therapy for osteoporosis and the development of novel anti-osteoporosis treatments may become key research focuses in the future.

## 4 Discussion

### 4.1 Current situation

The findings of this study indicate that the volume of relevant literature in the area of drug treatment for osteoporosis has generally increased over time. From 2014 to 2022, there was a period of rapid growth, with the annual number of published papers rising from 219 in 2015 to 352 in 2022, highlighting a notable surge in research interest and activity in osteoporosis drug treatment. From 2022 to 2024, this trend began to stabilize with a slight decline: after peaking in 2022, the number of annual publications slightly decreased in 2023 and 2024, yet remained relatively high (335 and 306 papers, respectively), suggesting that the research area has reached a more mature stage. In summary, research into drug treatments for osteoporosis has undergone significant growth in the last decade and has now reached a point of stability, indicating notable advancements and continued attention in the field.

National analysis reveals that the United States and China are leading in the number of published papers in this domain. The cooperative relationship between these two countries has been relatively close and has played a crucial role in advancing research in this area. Regarding research institutions, the University of Toronto in Canada has published the most papers, while the Catholic University of Australia leads in citation counts, reflecting the high academic standing of these institutions. Among individual authors, Maria Luisa Brandi from Italy has published the most articles in this field, with 14 articles to her name. She holds significant international influence in endocrinology and bone metabolic diseases, particularly contributing to the standardization of diagnosis and treatment for MEN1, osteoporosis, and parathyroid disorders (8, 9). Furthermore, co-citation analysis reveals that Kanis JA from the UK has had her article cited 948 times, making her the most cited author. As a leading authority in osteoporosis research, she has greatly improved fracture risk prediction and clinical management efficiency through the development of the FRAX tool, advancing diagnostic standards, and conducting global epidemiological studies. These findings underscore the substantial contributions and central role of these two authors in the field (10, 11). In terms of journals, Osteoporosis International is prominent in both citation frequency and total citations, underlining its significant influence in the field.

A deeper evaluation of the most highly cited papers is essential to understand their impact on shaping the field of osteoporosis pharmacological treatment. These key publications often serve as milestones that not only advance scientific knowledge but also guide future research directions. By analyzing the annual publication trends from 2015 to 2024, we observed a remarkable turning point in 2019, when the number of publications increased by 13.9% compared with the previous year (+30 articles), exceeding 240 publications for the first time. One possible explanation for this surge is the publication of a landmark article by Compston in The Lancet (6). Authored by one of the most authoritative experts in the osteoporosis field, this work provided a comprehensive synthesis of therapeutic progress and offered critical insights for both clinical practice and future research directions. Its influence is reflected not only in the exceptionally high citation frequency but also in its role as a pivotal reference that bridged past advances with emerging strategies. This milestone publication has had a profound impact on subsequent research output, underscoring how highly cited articles can shape the trajectory of the field.

### 4.2 Research hotspots

Through co-citation and co-occurrence analysis of literature and keywords, it is evident that the primary research clusters focus on targeted drug delivery mechanisms (such as extracellular vesicles and drug delivery systems), alongside evaluating the clinical efficacy and safety of various drugs for osteoporosis treatment (including romosozumab, denosumab, and bisphosphonates). Additionally, research on drug management related to osteoporosis has been extensively reported, covering topics such as medication adherence, breast cancer, drug-related osteonecrosis, and bone mineral density.

#### 4.2.1 Targeted drug delivery mechanism

In recent years, the focus of osteoporosis drug research has increasingly shifted toward targeted drug delivery systems, particularly the use of extracellular vesicles (EVs) and drug delivery mechanisms. EVs, as natural nanocarriers, possess excellent biocompatibility and targeting capabilities, allowing them to effectively concentrate drugs in specific tissues while minimizing adverse effects on non-target areas (12, 13). As a result, researchers have proposed strategies to enhance the drug-loading efficiency of EVs, improve their penetration abilities, and boost targeting potential, with the goal of understanding the molecular mechanisms behind drug treatment for osteoporosis at the molecular level (14, 15). These approaches allow for more precise drug release, optimizing therapeutic effects while reducing side effects, ultimately enhancing the overall efficacy of treatments.

Currently, research on molecular mechanisms primarily targets potential therapeutic drug targets, especially molecular pathways linked to bone formation and resorption. For instance, the Wnt/β-catenin pathway (16), the RANK/RANKL/OPG signaling pathway (17), and others have been shown to play critical roles in the development and progression of osteoporosis. Targeting these pathways not only aids in osteoporosis treatment but also offers a theoretical foundation for developing targeted therapies that are both more effective and have fewer side effects. Future research should further investigate the optimization of extracellular vesicles and other drug delivery systems, laying the groundwork for the development of new, more effective therapeutic drugs.

#### 4.2.2 Clinical efficacy and safety of different drugs in osteoporosis treatment

The focus of osteoporosis drug research is now shifting toward evaluating the clinical efficacy and safety of various treatments. While calcium and vitamin D supplements remain widely used in basic osteoporosis treatment, their long-term effectiveness is limited. Literature reports indicate that their therapeutic impact on preventing and treating osteoporosis is not ideal (18, 19). Consequently, there is an urgent need for more effective drugs with fewer side effects. Clinical studies on osteoporosis medications such as romosozumab, denosumab, and bisphosphonates have increased in recent years, showing substantial therapeutic effects in clinical settings.

Romosozumab inhibits bone resorption by targeting the RANKL signaling pathway (20), denosumab directly blocks RANKL function (21), and bisphosphonates enhance bone mineral density by inhibiting osteoclast activity (22). These drugs have demonstrated favorable therapeutic outcomes in various patient groups (23, 24), significantly reducing the risk of fractures. However, despite preliminary clinical data supporting their safety and efficacy, large-scale, long-term studies are still necessary to further confirm their effectiveness and safety. Thus, future research should focus on the long-term effects and potential side effects of these drugs to provide more reliable clinical treatment options for osteoporosis.

#### 4.2.3 Drug management in osteoporosis

Osteoporosis pharmacological therapies can broadly be categorized into anabolic agents, which stimulate bone formation, and catabolic (antiresorptive) agents, which inhibit bone resorption. Among the anabolic therapies, teriparatide (PTH 1-34) and abaloparatide are well-established and widely used in clinical practice, with proven efficacy in increasing bone mineral density and reducing fracture risk. In contrast, antiresorptive therapies, such as bisphosphonates and denosumab, act by suppressing osteoclast-mediated bone resorption. This classification provides a clearer framework for understanding the therapeutic landscape and reflects the dual strategies that underpin current treatment paradigms.

Research on osteoporosis drug management is currently centered on optimizing treatment regimens and improving patient adherence to medication. Although there are several drugs available for osteoporosis treatment, such as bisphosphonates, denosumab, and romosozumab, there remains a gap in clinical outcomes. One critical factor is medication adherence, as many patients fail to take their medication consistently due to the burden of long-term treatment, side effects, or lack of trust in treatment efficacy, directly affecting drug effectiveness (25, 26). Therefore, enhancing medication compliance is essential for improving therapeutic outcomes.

Recent studies have shown that the clinical efficacy of monotherapy in osteoporosis treatment, particularly with teriparatide, is often inferior to that of combination therapies. Evidence from recent meta-analyzes indicates that combining teriparatide with bisphosphonates or denosumab provides superior outcomes in terms of bone mineral density and fracture risk reduction compared to teriparatide alone. For example, Sun et al. (27) demonstrated that the combination of teriparatide and denosumab is more effective than teriparatide monotherapy in postmenopausal osteoporosis. Similarly (27), Jin et al. (28) and Chen et al. (29) highlighted the enhanced efficacy of combination therapies, showing improved bone health and lower fracture rates (28, 29).

The synergistic effect of anabolic and anti-resorptive agents, such as teriparatide with bisphosphonates or denosumab, provides a more comprehensive approach by stimulating bone formation while inhibiting bone resorption. These findings suggest that combination therapy offers a more robust and effective strategy for osteoporosis treatment compared to monotherapy. Recent advances in osteoporosis treatment have highlighted the role of protein- and peptide-based therapeutics, as discussed in the review “Emerging Protein and Peptide Therapeutics for Osteoporosis: Advances in Anabolic and Catabolic Treatments” (30). These therapies, including parathyroid hormone analogs and RANKL inhibitors, target both bone formation and resorption. This aligns with the trends identified in our bibliometric analysis, which suggests an increasing focus on combination therapies that integrate anabolic and antiresorptive agents for enhanced clinical outcomes.

Moreover, certain breast cancer medications, such as estrogen receptor inhibitors, can contribute to the development of osteoporosis, increasing the risk of fractures and related complications (31). The side effects of these drugs complicate the treatment and management of osteoporosis patients. Although bisphosphonates and denosumab are common anti-osteoporosis treatments, they may lead to serious side effects, such as drug-induced osteonecrosis, necessitating cautious use (32).

Monitoring bone mineral density is a key method for assessing the therapeutic effects of osteoporosis treatments. Regular bone density evaluations provide clinicians with accurate data, allowing for adjustments to treatment plans based on patients’ specific needs and improving treatment outcomes (33). Therefore, future research should focus on managing drug side effects, improving medication adherence, and developing effective bone density monitoring strategies, thus providing a more scientific foundation for personalized osteoporosis treatment.

### 4.3 Future trends

Using keyword analysis from Citespace, recent and emerging keywords have been identified, shedding light on potential future research directions. A closer examination suggests that future developments may focus on the specific mechanisms of molecular targeted therapies and the investigation of novel therapeutic targets. In this context, extracellular vesicles (EVs) and exosomes, as significant biological carriers, are gaining attention in osteoporosis research. Exosomes, in particular, are capable of carrying various bioactive molecules such as proteins, lipids, and RNA, which can influence bone cell functions, promote bone regeneration, and inhibit bone resorption (34). Numerous systematic animal studies have been carried out to explore the potential applications of extracellular vesicles (EVs) in osteoporosis treatment. A recent systematic review and meta-analysis published in August 2025 analyzed six independent studies involving a total of 92 animal models for osteoporosis, conducted between 2020 and 2024. Using C57BL/6 mice or SD rats with ovariectomies as primary models, the research established that EVs derived from dietary sources such as milk, yam, and oyster could significantly enhance bone mineral density (BMD) and trabecular bone thickness (Tb.Th), while also decreasing markers of bone resorption, namely β-CTX and TRACP-5b (35). Moreover, a study published in April 2025 on the “Zn-Cu alloy scaffold combined with low-intensity pulsed ultrasound” further confirmed that exosomes emitted by Schwann cells could markedly elevate the expression of osteogenic genes (such as COL1 and OCN) in bone marrow stromal cells (BMSCs) sourced from osteoporotic rats. This indicates that the integration of EVs with physical stimulation may lead to a synergistic effect on bone regeneration (36). To date, there have been no registered or published Phase I-III clinical trials focused on osteoporosis. The application of EVs for osteoporosis treatment remains in the animal study phase and has not yet progressed to human clinical trials.

The use of extracellular vesicles in drug delivery, especially when combined with nanoparticles, holds substantial promise. Nanoparticles, due to their smaller size and greater surface area, improve drug bioavailability, targeting, and stability (37). By encapsulating drugs within exosomes or nanoparticles, the degradation and adverse reactions of drugs in the body can be minimized, while enhancing targeted delivery to bone tissue, thereby improving the effectiveness of osteoporosis treatments. Targeted delivery of nanoparticles to bone tissue could not only stimulate bone regeneration but also aid in restoring bone density by regulating osteogenic differentiation. Various nanoparticle systems have been extensively verified using multiple animal models for osteoporosis treatment. Numerous types of nanocarriers designed for targeting bone tissue (including PLGA, chitosan, liposomes, and hydroxyapatite nanorods) have been widely documented (38). However, as of August 2025, no clinical trial registrations or results pertaining to nanomedicines listed for “osteoporosis” as an indication have been identified. Before these treatments can be implemented in clinical settings, comprehensive animal safety and toxicology studies, as well as GMP-level process validation and preliminary human trials, must still be undertaken.

Simultaneously, research into the molecular mechanisms of osteoporosis has revealed the crucial roles of apoptosis and inflammatory responses in its development. Studies indicate that osteoporosis is closely linked to the overactivity of osteoclasts and the reduced activity of osteoblasts (39). Apoptosis impacts bone remodeling by regulating the survival of osteoclasts and osteoblasts. Exosomes can modulate this process by inhibiting osteoclast overactivity through apoptosis regulation and promoting osteoblast survival and differentiation by reducing inflammatory responses, thus improving bone mineral density and encouraging bone regeneration (40). In recent years, developing targeted therapies focused on apoptosis mechanisms has emerged as a key strategy for enhancing osteoporosis treatment.

Inflammation plays a critical role in the initiation and progression of osteoporosis. Chronic inflammation can increase osteoclast activity through the release of pro-inflammatory cytokines such as IL-6 and TNF-α, exacerbating bone resorption and worsening osteoporosis (41). Researchers are exploring methods to suppress inflammatory responses in bone tissue by utilizing anti-inflammatory factors within exosomes, which could reduce bone resorption and improve bone regeneration and repair (42). This approach opens up new molecular targets for treating osteoporosis.

As molecular targeted therapies continue to evolve, future research may focus on integrating these advanced carrier systems with targeted molecular treatments, aiming for personalized approaches to osteoporosis treatment through precise drug delivery. By targeting molecular mechanisms involved in osteogenic differentiation and inhibiting bone resorption, coupled with advanced delivery systems like extracellular vesicles and nanoparticles, the therapeutic efficacy of osteoporosis treatment can be optimized. This approach would minimize side effects, offering a safer and more effective treatment for clinical use.

Looking toward the future, emerging therapeutic candidates such as PEPITEM (Peptide Enriched in TGF-β Induced Messenger) represent promising directions in osteoporosis management. PEPITEM has attracted increasing attention for its potential to modulate immune–bone interactions and may open new avenues for mechanism-driven therapies. Integrating these novel agents with existing anabolic and catabolic strategies highlights the ongoing transition toward more personalized and targeted approaches in osteoporosis treatment (43).

Thus, the future direction of research will likely center on discovering new therapeutic targets and deepening understanding of their molecular mechanisms, fostering the development of personalized, targeted, and precise treatments for osteoporosis.

In summary, the future of osteoporosis drug therapy will extend beyond traditional treatments to incorporate molecular targeted therapies and advanced drug delivery systems. By utilizing multiple mechanisms, these therapies aim to maximize improvements in bone density, structure, and the overall quality of life for patients, offering a more comprehensive solution for managing osteoporosis.

### 4.4 Study limitations

This research is limited to English-language literature sourced from the Web of Science core database. Consequently, relevant studies published in other languages or databases, such as CNKI or Wanfang, may have been excluded, particularly those related to traditional medicines like traditional Chinese medicine from non-English-speaking regions. However, the Web of Science, being a highly authoritative database, includes rigorously selected journals that comprehensively represent high-quality research on osteoporosis treatment, thus providing a reliable reflection of the core advancements in this field. Additionally, the time frame for this study spans from 2014 to 2024, focusing on research trends over the past decade. This selection may overlook earlier influential literature that shaped the understanding of mechanisms in osteoporosis research. Furthermore, the CiteSpace software generates a knowledge graph by analyzing citation relationships in the literature. While it offers an objective view of the structure and evolutionary trends in the field, it cannot deeply analyze the specifics of the studies (such as experimental designs or therapeutic differences). As a result, the interpretation of findings still depends on the subjective judgment of the researchers. Future studies could improve the breadth and depth of analysis by incorporating data from multiple databases, including multilingual sources, and combining quantitative and qualitative research methods.

### 4.5 Summary and future directions

This manuscript provides a detailed bibliometric analysis of drug treatment research for osteoporosis from 2015 to 2024, highlighting the current state and future trends in the field. The findings demonstrate that research on osteoporosis drug treatments continues to grow in both the number of publications and citations, with the United States and the University of Toronto at the forefront. Current research primarily focuses on the drug delivery mechanisms, clinical efficacy and safety of various treatments, and the management of osteoporosis medications. Emerging keywords such as “extracellular vesicles,” “exosomes,” “apoptosis,” and “bone regeneration” suggest that future studies may delve deeper into the specific mechanisms of molecular-targeted drugs and explore potential therapeutic targets. Notably, research on drug delivery systems utilizing nanotechnology and the regulation of bone metabolism through apoptosis and inflammatory responses may become more prominent. These developments could provide novel directions for the precise and personalized treatment of osteoporosis.

## Data Availability

The original contributions presented in this study are included in this article/supplementary material, further inquiries can be directed to the corresponding author.
